# High Prevalence of Insecure Attachment in Patients with Primary Hypertension

**DOI:** 10.3389/fpsyg.2016.01087

**Published:** 2016-08-03

**Authors:** Elisabeth M. Balint, Manuela Gander, Dan Pokorny, Alexandra Funk, Christiane Waller, Anna Buchheim

**Affiliations:** ^1^Department of Psychosomatic Medicine and Psychotherapy, University HospitalUlm, Germany; ^2^Institute of Psychology, University of InnsbruckInnsbruck, Austria

**Keywords:** primary hypertension, attachment representation, somatic disease, cardiovascular response, cardiovascular reactivity

## Abstract

Hypertension is a major cardiovascular (CV) risk factor and is predicted by heightened CV reactivity to stress in healthy individuals. Patients with hypertension also show an altered stress response, while insecure attachment is linked to a heightened stress reactivity as well. This is the first study aiming to assess attachment representations in patients with primary hypertension and to investigate their CV responses when their attachment system is activated. We studied 50 patients (38 men, 12 women) with primary hypertension. The Adult Attachment Projective Picture System (AAP), a widely used and validated interview, was performed to measure the patients' attachment representations, and to activate their attachment system. Blood pressure and heart rate were measured after 10 min at rest prior to and directly after the AAP interview. Mood and state anxiety were assessed using the Multidimensional Mood State Questionnaire (MDBF) and the State Trait Anxiety Inventory-State (STAI-S) before and after the experiment. The prevalence of insecure attachment (dismissing, preoccupied, unresolved) in hypertensive patients was predominant (88%), while in non-clinical populations, only about 50% of individuals had insecure attachment patterns. Blood pressure (*p* < 0.001), heart rate (*p* = 0.016), and rate pressure product (*p* < 0.001) significantly increased in response to the attachment interview. Secure attached patients showed the highest rise in systolic blood pressure (*p* = 0.020) and the lowest heart rate compared to the other attachment groups (*p* = 0.043). However, attachment representation showed no significant group or interaction effects on diastolic blood pressure (DBP) and rate pressure product. Insecure attachment was highly over-represented in our sample of patients with primary hypertension. Additionally, a robust CV response to the attachment-activating stimulus was observed. Our data suggest that insecure attachment is significantly linked to primary hypertension, which implies the need for further investigations to evaluate attachment insecurity as a possible risk factor for hypertension.

## Introduction

Arterial hypertension represents a major cardiovascular (CV) risk factor worldwide and accounts for approximately 9.4 million deaths per year. Epidemiological studies demonstrate that about 30–45% of the adult population is affected by arterial hypertension (Mancia et al., 2013).

Primary hypertension refers to a group of patients with no secondary causes like pheochromocytoma, renal failure, aldosteronism, or renovascular diseases and it accounts for almost 95% of all cases of hypertension (Carretero and Oparil, 2000). Among other factors, it is predicted by heightened (CV) reactivity to stress (Matthews et al., 1993, 2004; Carroll et al., 2013). The magnitude and patterns of CV stress reactivity differ highly between individuals (Lawler et al., 2001), and are only partly explained by genetic factors (Wu et al., 2013). Animal studies showed that variations in maternal care altered neuroendocrine (Liu, 1997) as well as CV stress responses in rats (Loria et al., 2010). A prospective study in humans showed that the perception of parental care predicted health status in midlife (Russek and Schwartz, 1997). Further research focused on attachment to provide a comprehensive framework for understanding how early experiences might contribute to disease risk. Potential pathways include changes in physiological stress systems, immunity, health-, and risk behavior (Maunder and Hunter, 2001, 2008, 2009; Gander and Buchheim, 2015). Based on these concepts, attachment has been further evaluated in a broad context using different measures of attachment, mostly self-report. Attachment anxiety was found to have a strong positive association with psychosomatic complaints (Wulf et al., 2012) and insecure attachment was associated with higher levels of somatization (Waller et al., 2004; Waldinger et al., 2006). A high prevalence of insecure attachment has been shown in patients with somatic and somatoform diseases: 70% in diabetes (Ciechanowski et al., 2001), 72% in patients with ulcerative colitis (Maunder et al., 2000), and 74% in somatoform disorders (Waller et al., 2004).

In the field of hypertension research, a large epidemiological study reported a positive association between insecure anxious attachment style and hypertension. In 5645 participants from the US National Comorbidity Survey Replication, attachment style was assessed by self-report questionnaires measuring the dimensions of secure, anxious, and avoidant attachment (Hazan and Shaver, 1987), while life-time depression, anxiety, and alcohol- or substance related disorders were evaluated by clinical interviews. Life-time history of high blood pressure (BP) was also assessed. The association between high BP and anxious attachment ratings remained positive even after controlling for the assessed psychiatric disorders (McWilliams and Bailey, 2010). However, this cross-sectional study cannot contribute to causality or pathological pathways. Until now, only few studies investigated BP responses to attachment related stressors and all were performed in healthy individuals. Gallo and Matthews (2006) investigated ambulatory BP in adolescents and showed a positive association between systolic and diastolic BP and anxious attachment during interactions with friends, whereas avoidant attachment correlated with an augmented diastolic blood pressure (DBP) during social conflict. Feeney and Kirkpatrick (1996) demonstrated corresponding results in couples, while Kim showed a reduced rate-pressure product (RPP) reactivity in more avoidant individuals (Kim, 2006).

As mentioned before, these studies measured attachment by self-reports. In adults, there are two major approaches to assess attachment styles or attachment representations, namely self-report measurements and narrative methods. Self-report measures are a product of subjective and conscious thoughts and mostly differentiate between secure and insecure avoidant or anxious attachment (Ravitz et al., 2010), while narrative methods allow analyzing representations of attachment including defensive processes (e. g., deactivation, repression). Defensive processes serve to protect against distressful and overwhelming emotions. These measures differ between three modes of insecure attachment representations (dismissing, preoccupied, and unresolved trauma). As research findings demonstrate a significant association between hypertension and lower affect expression signaling a lower level of emotional awareness (Jorgensen et al., 1996; Mann and James, 1998; Consoli et al., 2010). Mann (2012) suggested to focus on repressed and unaware emotions especially in hypertensive patients. Since the Adult Attachment Projective Picture System (AAP; George and West, 2012) includes the analysis of unconscious defensive processes to assess attachment representations, the present study used this instrument as a reliable and valid narrative attachment measure. It is conceptualized that the seven attachment-related scenes depicting solitude, separation, illness, abuse, and death activate the attachment system of an individual and thus produce potential stress and physiological arousal. The participants are asked to tell a story to each picture using a series of standardized prompts. The feasibility of the AAP measure as an attachment activating stimulus has been proven in diverse experimental settings in clinical and non-clinical groups (Buchheim et al., 2006, 2008, 2012).

Attachment representations are categorized into four groups: secure, insecure-dismissing, insecure-preoccupied, and unresolved trauma. According to the language of narrative measures, and here especially to the classification rules in the AAP, secure (F) individuals show a high level of agency, connectedness, and synchrony in attachment relationships in their narratives. If they use defensive strategies, they serve a more flexible integration at the representational level (high agency, e.g., thinking processes). Insecure-dismissing (Ds) individuals or preoccupied (E) individuals are characterized by functional or absent relationships in the stories. Those with dismissing representation rather use “deactivation” (represented e.g., by rejection, power, or achievement), whereas those with a preoccupied representation use a high amount of “cognitive disconnection” as a characteristic defense (represented e.g., by conflicts, vagueness, or anger). Unresolved (U) individuals are overwhelmed by topic related to attachment related trauma (e.g., danger, isolation, fear, or threat) and loss with no indications of the character's capacity to act, like protection from frightening and dangerous situations and no internalized available attachment figure providing comfort and security (Bowlby, 1980; George and West, 2012). Since secure adults are able to reflect and integrate positive as well as negative attachment related experiences (West and Sheldon-Keller, 1994), researchers conceptualized that secure attachment can have a regulatory effect on affective and physiological responses to distress as these individuals are more flexible in their emotional expressions (Gross, 2007; Vrtička and Vuilleumier, 2012). Studies with children have shown that the more adaptive strategy of securely attached children leads to a lower heart rate (HR) and cortisol response during the Strange Situation Procedure (Dozier and Kobak, 1992; Hill-Soderlund et al., 2008; Gander and Buchheim, 2015), while adults rated as securely attached by the Adult Attachment Interview (AAI) demonstrated a low HR response to the AAI (Beijersbergen et al., 2008) and only a small increase of skin conductance during a marital conflict task (Roisman, 2007).

In contrast, individuals with an insecure attachment classification developed defensive strategies that modulate difficult and anxious attachment related experiences (George and West, 2012). In response to attachment-related situations, insecure-dismissing individuals employ defensive processes that emphasize distance in relationships; they usually suppress their emotions and mask their negative affect (Dozier and Kobak, 1992). Studies demonstrated that in conflicting tasks with primary caregivers or partners, dismissing individuals exhibited an increased HR. This suggests that their repressive coping style was ineffective in regulating their increased level of physiological arousal when directly confronted with attachment-related situations (Kim, 2006; Beijersbergen et al., 2008).

Insecure-preoccupied individuals have experienced inconsistent availability of their caregivers, leading to negative affect, irritability in relationships, and hyper-vigilance about gaining and maintaining time and attention from their attachment figures (George and West, 2012). Some studies showed an enhanced acceleration in HR, for example while conversing with their partners in a conflict interaction task that might mirror their emotional over-involvement (Dozier and Kobak, 1992; Roisman, 2007; Holland and Roisman, 2010).

Disorganized or unresolved individuals exhibit conflicted, disoriented, or contradictory behaviors indicating an inability to maintain a coherent attachment strategy when confronted with emotional distress, while organized or resolved subjects are able to cope with this distress successfully. The absence of predictable emotional strategies in the unresolved group often lead to the most poorly managed emotions, and subsequently increase the risk for adjustment difficulties and psychopathology (Kobak and Madsen, 2008; Weinfield et al., 2008; Bakermans-Kranenburg and Van IJzendoorn, 2009). Several studies in infants have demonstrated that this attachment pattern is associated with tremendous stress during attachment-related stressors as indicated by the highest cortisol levels and HR accelerations of all four attachment groups (Hertsgaard et al., 1995; Willemsen-Swinkels et al., 2000; Bernard and Dozier, 2010). In the interview setting, this attachment pattern is characterized by a breakdown of defensive and coping strategies especially while talking about traumatic experiences like loss and abuse (Buchheim and George, 2011). The few studies in adults have shown inconsistent results. Beijersbergen et al. (2008) reported no differences in CV responses between resolved (secure, dismissing, preoccupied) and unresolved individuals during the AAI, whereas Stanley (2006) found a higher arousal in HR in the unresolved group while watching emotional video scenes on separation and reunion.

To date, no study has investigated the interplay between attachment representations in hypertensive patients and physiological arousal when the patients' attachment system is activated. In sum the aim of this study was to examine the potential of attachment theory to be added into the bio-psycho-social model of medicine (Engel, 1980). Here, attachment representations might contribute as psychosocial factors to the development of hypertension. The attachment model provides an insight into repeated crucial interactions between infants and their attachment figures that might result in stable patterns, measured by the internal working models of attachment in adults. These internal models of attachment have an important impact on stress response, interpersonal resources, and vulnerability to illness (Maunder and Hunter, 2001, 2008). The main purpose of this study was to determine attachment representations and CV parameters in response to attachment-related stress in hypertensive patients. Based on the previously mentioned CV differences among the attachment groups we hypothesized that (1) the majority of our sample shows insecure attachment patterns, (2) we observe a CV response to the attachment activating stimuli (AAP task), and (3) that we find differences in these CV responses among the attachment groups.

## Materials and methods

### Participants

Recruitment was realized at the Cardiologic Outpatient Clinic at the Ulm University Hospital. We recruited patients with a clinical diagnosis of primary hypertension treated with at least one anti-hypertensive drug. Further inclusion criteria were age between 18 and 80 years and sufficient knowledge of the German language. We excluded patients with thyroid dysfunction, heart insufficiency with ejection fraction < 35%, severe valvular stenosis or insufficiency, end-stage renal disease with regular dialysis, current alcohol or drug abuse, and patients who received renal denervation for treatment of uncontrolled hypertension. Patients with cognitive deficits following stroke, current psychosis, and dementia were excluded if they showed obvious deficits in communication. Due to planned examination of blood biomarkers, we further excluded patients with chronic rheumatic diseases and patients with cortisol intake due to other comorbidities during the last 3 months. The 71 patients fulfilling in- and exclusion criteria and willing to participate in our study were screened for post-traumatic stress disease (PTSD) using the Post-traumatic Diagnostic Scale (PDS; Griesel et al., 2006). We excluded 21 patients with positive screening due to possible PTSD-related alterations in stress reactivity (Pineles et al., 2013). Thus, 50 patients participated in our study.

### Procedure

The study protocol was approved by the ethic committee of Ulm University and was conducted according to the Helsinki Declaration with written informed consent from all subjects. Subjects were invited individually to the lab at 02:00 p.m. They were instructed to have their lunch at 12:00 a.m. on the day of the appointment. After arrival, medication and medical history were assessed. Thereafter, the patients were asked to lie down and the whole procedure was conducted while the patient was lying in order to minimize artifacts on BP via arm or leg movements. Electrodes and BP cuffs were attached to the chest and both upper arms, respectively. BP was measured using a Sphygmomanometer (Model VS-1500N, serial number 50000071, Fukuda Denshi Co Ltd., Tokyo, Japan) simultaneously on the right and the left upper arm at different time points as described below, while ECG was recorded continuously using NeXus-10 wireless physiological monitoring (serial number 0928100209, Mindmedia, Oldenzaal, Netherlands).

After filling out questionnaires on mood and anxiety (MDBF and STAI-S, see description below) and a resting period of 10 min, BP was measured twice. Thereafter, the AAP was performed. Immediately after the last picture, BP was measured once and the questionnaires MDBF and STAI-S were administered again.

### CV data analyses

BP was calculated as the mean of right and left arm measurement. Resting BP was calculated as the mean of the two consecutive measurements at rest. HR was calculated as the mean of the minute before BP measurement. RPP was calculated as the product of HR and systolic blood pressure (SBP; Folkow and Ely, 1998).

### Assessment of attachment with the AAP

The AAP (George and West, 2012) is a measure to assess adult attachment representations by using a set of eight picture stimuli (one neutral, seven attachment scenes). The participant was asked by the interviewer using standardized questions (What happens in the scene, What are the characters thinking or feeling? What might happen next?) to tell a story to each picture. Narratives were audio-recorded and verbatim transcripts were analyzed. Content and defensive processes were coded in each story: deactivation (avoidance), cognitive disconnection (ambivalence), and segregated systems (attachment fear and its resolution). The AAP designates four attachment classifications based on the analysis of the coding dimensions across the entire set of seven attachment stories. A number of studies have tested psychometric properties of the AAP and demonstrated satisfying evidence of concurrent validity with the AAI [concordance rates for the four-group classifications were 90%, κ = 0.84 and for the two groups (secure vs. insecure) even 97%, κ = 0.89], test–retest reliability after 3 months (84%, κ = 0.78), inter-judge reliability (concordance rate were 90%, κ = 0.85), and discriminant validity in clinical and healthy adult samples (Benoit et al., 2010; Buchheim and George, 2011; George and West, 2011, 2012; Buchheim et al., 2014). Moreover studies on the discriminant validity showed no influence of social desirability, verbal intelligence, and socio-demographic variables on the AAP classifications (George and West, 2012; Gander et al., 2016). All AAP protocols in our study were analyzed by highly experienced judges (AB, MG), one of them certified to train the AAP in the German language (AB). Seven interviews could not be coded for technical reasons. These patients were excluded from analyses of attachment representations.

### Assessment of mood and anxiety

For assessing mood, we used the German version of the Multidimensional Mood State Questionnaire (MDBF; Steyer et al., 1997). It consists of 12 items rated on a five-point Likert scale and measures three subscales (good–bad mood, alertness–tiredness, and calmness–restlessness). These scales are summed up, yielding a score between 4 and 20, with higher scores indicating better mood, higher alertness, and calmness. Internal consistency of the subscales within our study was satisfying with a Cronbach's alpha between 0.665 and 0.725 for each subscale at each time point.

State anxiety was assessed with the STAI-S (State Trait Anxiety Inventory-State; Laux et al., 1970; Spielberger et al., 1970). Twenty items are rated on a four-point Likert scale and summed up, yielding a score between 20 and 80. Higher scores reflect higher anxiety. Internal consistency of the scale was high with a Cronbach's alpha of 0.815 (before the interview) and 0.912 (after the interview).

### Statistical analyses

Data were analyzed using SPSS statistical software version 22 (SPSS Inc., Chicago, Illinois, USA). Two patients had absolute arrhythmia due to atrial fibrillation during the experiment and were therefore excluded from analyses of HR and RPP.

Significance level was set at *p* < 0.05 (two-sided). For calculation of differences between groups, Mann–Whitney-U-test, Kruskal–Wallis test, Fisher's exact test, and one-sample Chi-square test were used where appropriate. Comparison of the means of patients to the means of healthy controls from the literature were conducted using one-sample *t*-test. Significances for the comparison of distribution of attachment classifications in our sample and a non-clinical European sample were calculated by two-sided exact binomial test and exact multinomial test. Shapiro–Wilk test rejected normal distribution of MDBF, but not of SBP, DBP, HR, RPP, and STAI-S, which were also visually normally distributed according to P–P plots. Therefore, related-samples Friedman's two-way analysis of variance by ranks was applied to test significant differences in mood and anxiety before and after the AAP interview. Concerning CV responses to the AAP, exact Sign-test was applied first to test for the direction of CV responses to the AAP, while ANOVA for repeated measures was applied to test significant differences of means before and after the AAP interview. To determine attachment groups and interaction effects, a four groups (attachment categories) by two time points (before and after the interview) ANCOVA was performed with each CV measurement as dependent variable. To control for potential confounders, age, sex, body-mass index, and number of anti-hypertensive drugs were entered as covariates.

## Results

### Sample characteristics

About three quarters of our patients were male (*N* = 32, 74%). Most of them were elderly (*M* = 65 years, *SD* = 10 years) and living in a partnership (*N* = 36, 88%). Hypertension was treated with maximal five anti-hypertensive drugs, with 15 (30%) of the patients taking two, 16 (32%) taking three and twelve (24%) taking four different anti-hypertensive drugs. Comorbidity was high: 17 patients (40%) had a history of myocardial infarction and 10 patients (23%) had diabetes. The most frequent number of diagnoses was six with a range between two and twelve and the most frequent number of total drugs was five with a range between two and 13. Data are shown in Table 1.

**Table 1:** **Patient characteristics for each attachment classification**.

**Attachment representation, *N* (%)**	**Secure 5 (12%)**	**Insecure-dismissing 7 (16%)**	**Insecure-preoccupied 18 (42%)**	**Unresolved trauma 13 (30%)**	**Total 43 (100%)**	***p*-values**
**SOCIOECONOMIC DATA**						
Male gender, *N* (%)	4 (80%)	3 (43%)	13 (72%)	12 (92%)	32 (74%)	0.10
Age (years), *M* ±*SD*	63.8 ± 12.7	62.6 ± 11.8	64.2 ± 9.6	66.6 ± 8.8	64.6 ± 9.8	0.85
Living in partnership, *N* (%)	5 (100%)	5 (83%)	15 (88%)	11 (85%)	36 (88%)	1.0
Higher school qualification, *N* (%)	3 (60%)	2 (29%)	10 (59%)	5 (38%)	20 (48%)	0.48
**MEDICAL DATA**						
Body Mass Index [kg/m^2^], *M* ±*SD*	32.4 ± 8.0	29.6 ± 7.2	28.4 ± 3.8	28.9 ± 6.1	29.2 ± 5.6	0.83
Number of anti-hypertensive drugs, *M* ±*SD*	3.4 ± 0.9	2.4 ± 1.0	2.7 ± 1.2	3.0 ± 0.7	2.8 ± 1.0	0.26
Total number of drugs, *M ± SD*	7.2 ± 2.3	5.9 ± 3.0	6.2 ± 2.7	8.5 ± 2.4	7.0 ± 2.8	0.066
History of myocardial infarction, *N* (%)	2 (40%)	3 (43%)	5 (28%)	7 (54%)	17 (40%)	0.52
Diabetes mellitus, *N* (%)	0 (0%)	2 (29%)	3 (17%)	5 (38%)	10 (23%)	0.34
Current smoking, *N* (%)	0 (0%)	1 (14%)	0 (0%)	2 (15%)	3 (7%)	0.25
Anti-depressant medication, *N* (%)	0 (0%)	0 (0%)	1 (6%)	3 (23%)	4 (9%)	0.33
Total number of diagnoses, *M* (*SD*)	5.6 ± 1.3	4.7 ± 2.4	5.3 ± 2.2	6.5 ± 2.7	5.6 ± 2.4	0.48
**PSYCHOMETRIC DATA**^a^						
MDBF good-bad mood, *M ± SD*	16.8 ± 2.8	17.7 ± 2.3	17.6 ± 1.6	17.2 ± 2.2	17.4 ± 2.0	0.85
MDBF alertness-tiredness, *M ± SD*	17.0 ± 2.9	16.2 ± 2.6	16.5 ± 2.3	13.6 ± 2.1	15.6 ± 2.7	0.014^*^
MDBF calmness-restlessness, *M ± SD*	16.4 ± 2.9	17.3 ± 1.1	15.9 ± 2.6	15.0 ± 2.8	16.0 ± 2.6	0.31
STAI-S state anxiety, *M ± SD*	34.2 ± 7.0	31.9 ± 4.2	31.9 ± 5.6	34.6 ± 4.7	33.0 ± 5.3	0.59

*Data are presented as frequencies (valid percent) and mean ± standard deviation. Significances for group differences were calculated by Kruskal-Wallis-test for continuous variables and by Fisher's Exact Test for nominal data*.

* *p < 0.05*.

a *Assessed at rest*.

Mood measured by MDBF was significantly better in our patients compared to a representative German sample (Hinz et al., 2012; *M* = 17.3, *SD* = 2.3 vs. *M* = 16.1, *SD* = 3.3, *p* < 0.001), while alertness (*M* = 15.6, *SD* = 2.7 vs. *M* = 15.2, *SD* = 3.2, *p* = 0.28) and calmness (*M* = 15.9, *SD* = 2.9 vs. *M* = 15.3, *SD* = 3.2, *p* = 0.14) did not differ significantly. Women had significantly lower state anxiety measured by STAI-S (*M* = 33.8, *SD* = 5.1 vs. *M* = 37.7, *SD* = 10.1, *p* = 0.030) than women of a normative sample. Differences for men on state anxiety compared to a normative sample were not significant (*M* = 32.9, *SD* = 6.0 vs. *M* = 34.4, *SD* = 9.3, *p* = 0.14).

### Distribution of attachment representation

**Figure 1:**
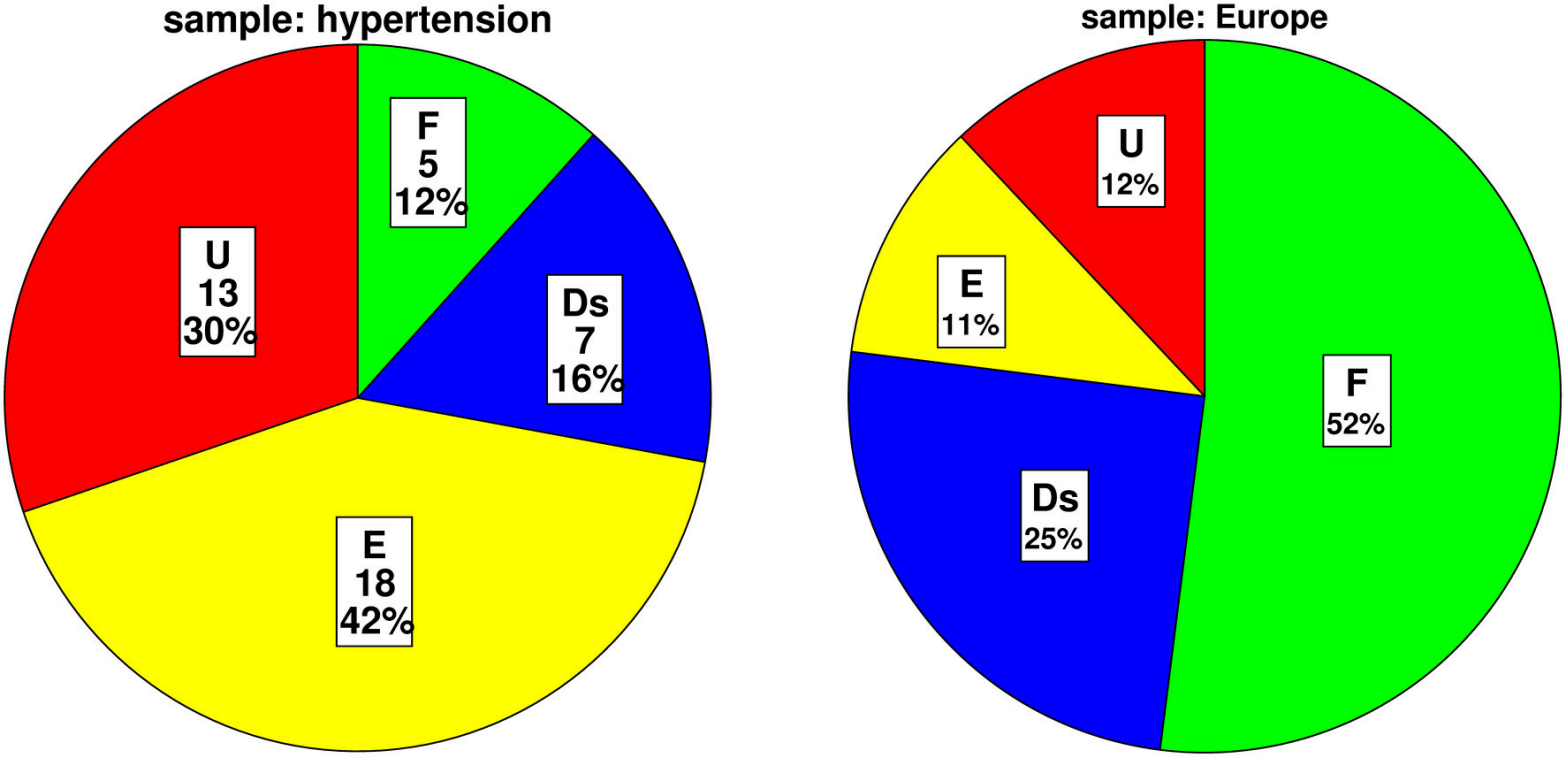
**Distribution of attachment classifications in our sample and a non-clinical European sample (Bakermans-Kranenburg and Van IJzendoorn, 2009)**. F, secure; Ds, dismissing; E, preoccupied attachment; U, unresolved trauma.

Of forty-three analyzable attachment interviews, we found five (12%; CI-95% = [4%; 25%]) patients with secure, seven (16%; [7%; 31%]) with dismissing, 18 (42%; [27%; 58%]) with preoccupied, and 13 (30%; [17%; 44%]) with unresolved trauma. Total prevalence of insecure attachment including dismissing, preoccupied and unresolved trauma was 88%; [75%; 96%]. Total prevalence of resolved attachment, which includes secure, dismissing and preoccupied attachment, was 70%; [56%; 83%], while unresolved attachment which refers to unresolved trauma was found in 13 (30%; [17%; 44%]) as described above. The distribution differs from a non-clinical European population (Bakermans-Kranenburg and Van IJzendoorn, 2009) with a significantly smaller proportion of secure attachment, a higher amount of preoccupied attachment and more unresolved trauma compared to the non-clinical sample. The distributions are visualized in Figure 1 and statistics are shown in Table 2.

**Table 2:** **Comparison of distribution of attachment classifications in our sample and a non-clinical European sample (Bakermans-Kranenburg and Van IJzendoorn, 2009)**.

**Attachment classification**	**Sample hypertension absolute frequency**	**Exact confidence interval CI-95% (%)**	**Sample hypertension relative frequency (%)**	**Sample Europe relative frequency (%)**	***p*-values**
Secure (F)	5	[4–25]	12	52	<0.001^***^
Insecure (Ds, E, U)	38	[75–96]	88	48	<0.001^***^
Dismissing (Ds)	7	[7–31]	16	25	0.220
Preoccupied (E)	18	[27–58]	42	11	<0.001^***^
Unresolved trauma (U)	13	[17–44]	30	12	0.001^**^
Total	43	[100]	100	100	<0.001^***^

*Significances were calculated by two-sided exact binomial test and in line “total” with exact multinomial test*.

** p<0.01;

*** *p<0.001*.

### Differences in patient characteristics between attachment groups

Patient characteristics for each attachment classification are shown in Table 1. Medical and socioeconomic data did not differ significantly between the four attachment classifications. Mood and state anxiety did not differ between groups except that individuals with unresolved trauma felt significantly more tired (*M* = 13.6, *SD* = 2.1) than the other groups (*M* = 16.2–17.0, *SD* = 2.3–2.9, *p* = 0.014).

Comparing the patients with unresolved trauma to those with organized representations (i.e., secure, dismissing, preoccupied), patients with unresolved trauma were treated with a higher number of total drugs (*M* = 8.5, *SD* = 2.4, *p* = 0.012) than patients with resolved attachment status (*M* = 6.3, *SD* = 2.7).

### Emotional responses to the AAP interview

Mood of participants and state anxiety of the participants did not change during the task. Alertness was significantly higher after the interview compared to rest, and calmness increased by trend (Table 3).

**Table 3:** **Mood and anxiety before and after the attachment interview**.

	**Before interview**	**After interview**	***p*-values**
MDBF good-bad mood	17.3 ± 2.3	17.1 ± 2.2	0.72
MDBF alertness-tiredness	15.6 ± 2.7	16.2 ± 2.4	0.034^*^
MDBF calmness-restlessness	15.9 ± 2.9	16.5 ± 2.6	0.068
STAI-S state anxiety	33.1 ± 5.8	33.4 ± 7.5	0.88

*Data are presented as mean ± standard deviation. Significances were calculated with related-samples Friedman's two-way analysis of variance by ranks. N = 48 for MDBF good-bad mood and STAI-S, N = 47 for MDBF alertness-tiredness and calmness-restlessness*.

* *p < 0.05*.

Individuals with unresolved trauma showed the highest decline in state anxiety of all four groups (*M* = 34.6, *SD* = 4.7 before the interview, *M* = 33.0, *SD* = 4.6 after the interview; *p* = 0.058). Only this group showed a significant change in alertness with the highest increase of alertness during the interview (*M* = 13.6, *SD* = 2.1 to *M* = 15.5, *SD* = 2.4; *p* = 0.016).

### Cardiovascular responses to the AAP interview

**Figure 2:**
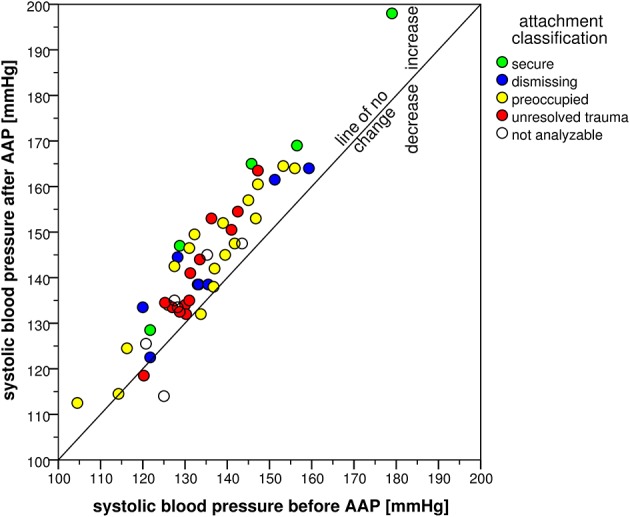
**Scatter plot of systolic blood pressure before and after the AAP**.

**Figure 3:**
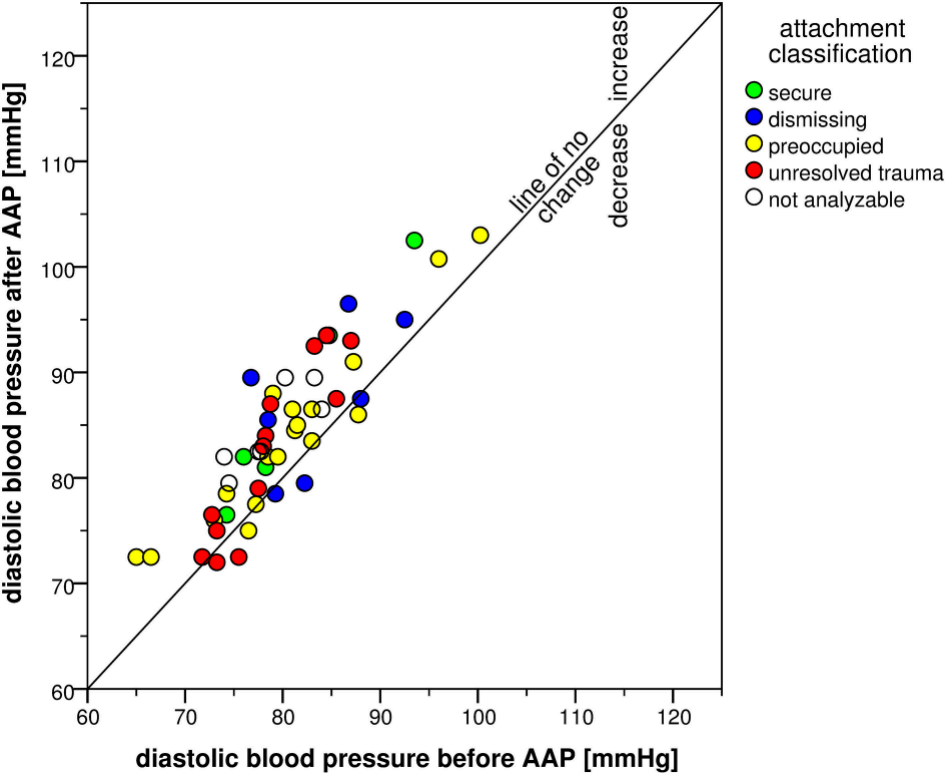
**Scatter plot of diastolic blood pressure before and after the AAP**.

**Figure 4:**
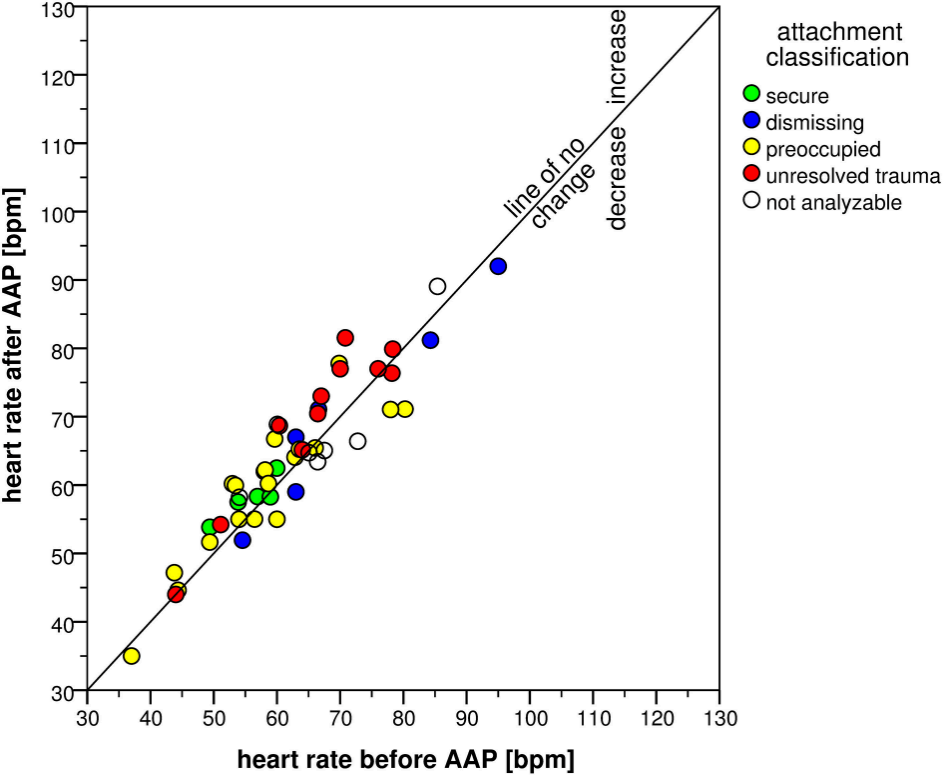
**Scatter plot of heart rate before and after the AAP**.

**Figure 5:**
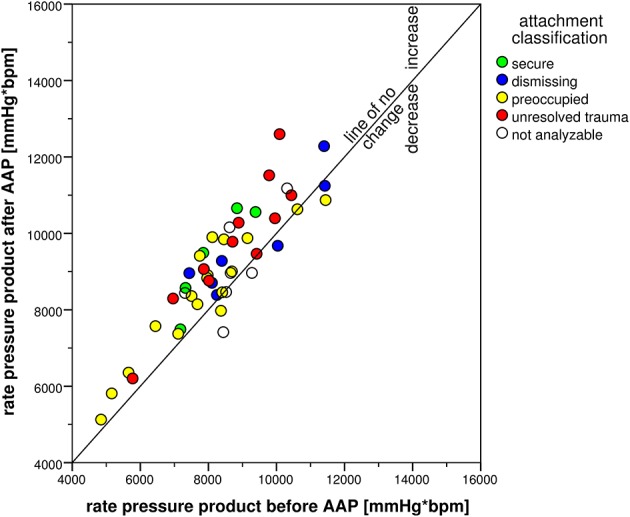
**Scatter plot of rate pressure product before and after the AAP**.

The attachment interview elicited significant CV responses with significant time effects for all CV measures (Table 4). Systolic blood pressure increased in 47 participants and decreased in only three participants (exact Sign-test: *p* < 0.001) with an average change of 8 mmHg (*SD* = 6 mmHg). Diastolic blood pressure showed similar results with an increase in 43 and a decrease in seven participants (exact Sign-test: *p* < 0.001) with an average change of 4 mmHg (*SD* = 4 mmHg). Heart rate showed a rise in 31 vs. a decline in 16 participants (exact Sign-test: *p* = 0.020) with an average rise of 2 bpm (*SD* = 4 bpm). Combining SBP and HR in the RPP resulted in an increase in 41 and a decrease in seven participants (exact Sign-test: *p* < 0.001) with a mean increase of 723 mmHg ^*^ bpm (*SD* = 725 mmHg ^*^ bpm). Though, SBP before starting the AAP showed a high range between 105 and 179 mmHg as well as DBP (between 65 and 100 mmHg) and HR (between 37 and 95 bpm), the increase after the AAP was consistent and considerable. This is visualized in Figures 2–5 which show the relation of CV parameters before and after the interview for each participant.

**Table 4:** **Cardiovascular responses to the attachment interview**.

	**Before AAP interview**	**After AAP interview**	***F***	***df***	***p*-values**	**Effect size, η^2^**
SBP (mmHg)	134.9 ± 13.1	143.1 ± 16.2	89.6	1.49	< 0.001^***^	0.65
DBP (mmHg)	80.2 ± 6.9	84.4 ± 7.8	65.4	1.49	< 0.001^***^	0.57
MAP (mmHg)	98.5 ± 8.2	104.1 ± 9.6	101.5	1.49	< 0.001^***^	0.67
HR (bpm)	62.7 ± 11.7	64.3 ± 11.4	6.3	1.47	0.016^**^	0.12
RPP (mmHg ^*^ bpm)	8417 ± 1508	9140 ± 1578	47.7	1.47	< 0.001^***^	0.50

*Data are presented as mean ± standard deviation. Significances are calculated with repeated measures ANOVA. SBP, systolic blood pressure; DBP, diastolic blood pressure; MAP, mean arterial pressure; HR, heart rate; RPP, rate pressure product. N = 50 for SBP and DBP; N = 48 for HR and RPP*.

** p < 0.01;

*** *p < 0.001*.

Further evaluation of the interview-associated CV responses by ANCOVA analyzing effects of the four attachment classifications showed no significant group effects except for HR [*F*_(3, 33)_ = 2.98, *p* = 0.046, η^2^ = 0.213]. Secure and preoccupied individuals showed a lower level of HR with 57 bpm (*SD* = 4 bpm) and 59 bpm (*SD* = 10 bpm) than dismissing (*M* = 69 bpm, *SD* = 14 bpm) and individuals with unresolved trauma (*M* = 68 bpm, *SD* = 11 bpm). Time × group interaction effects were not significant with the exception of *SBP* [*F*_(3, 35)_ = 3.72, *p* = 0.020, η^2^ = 0.242], referring to an interview-associated higher rise in secure individuals (15 mmHg, *SD* = 5 mmHg) than in dismissing (*M* = 8 mmHg, *SD* = 6 mmHg), preoccupied (*M* = 8 mmHg, *SD* = 5 mmHg) and individuals with unresolved trauma (*M* = 8 mmHg, *SD* = 5 mmHg).

## Discussion

### Findings

This is the first study investigating attachment representation in patients with hypertension. According to our hypotheses, we found a high prevalence of insecure attachment of 88%, which is almost twice as frequent as in a non-clinical sample (Bakermans-Kranenburg and Van IJzendoorn, 2009). The classification “secure” was underrepresented with only 12%, while 50% of secure attachment was found in the non-clinical sample mentioned before. At the same time, the classifications “insecure-preoccupied” and “unresolved trauma” were highly overrepresented with 42 and 30% compared to 11 and 12% of preoccupied attachment and unresolved trauma in the same non-clinical population (Bakermans-Kranenburg and Van IJzendoorn, 2009).

In other clinical populations that were examined using the AAI, the proportion of insecure attachment found in patients with somatoform disorders was 74% (Waller et al., 2004) and with mental disorders 77% (Bakermans-Kranenburg and Van IJzendoorn, 2009), which is similar to our results. Though working with a different concept of attachment, self-report measurements found comparably high proportions of insecure attachment styles in patients with somatic diseases, namely 70% in patients with diabetes (Ciechanowski et al., 2001) and 72% in patients with ulcerative colitis (Maunder et al., 2000).

The prevalence of unresolved trauma in our sample was with 30% almost as high as in clinical samples of patients with depression (41%) or addiction (43%), while patients with PTSD or borderline personality disorder show even more unresolved trauma (83 and 76%, respectively; Juen et al., 2013). However, in healthy controls, only 15% show unresolved trauma (Bakermans-Kranenburg and Van IJzendoorn, 2009; Juen et al., 2013).

This finding of a high proportion of insecure attachment representations in patients with hypertension and with other somatic diseases suggests that insecure attachment is not only related to mental disorders but also to bodily diseases. Insecure attachment patterns have been shown to disturb emotion regulation (Vrtička and Vuilleumier, 2012) and to modulate autonomic (Maunder and Hunter, 2008), neuroendocrine (Diamond and Fagundes, 2010; Gander and Buchheim, 2015), and immune (Vermeer et al., 2012; Fagundes et al., 2014) responses.

Our data consolidates the potential relevance of attachment for somatic homeostasis by showing that the AAP interview as an attachment activating stimulus elicits CV arousal, measured by significant BP, HR, and RPP responses.

Furthermore, our results demonstrated that the increase of alertness was highest in patients with an unresolved trauma. These individuals are unable to use defensive processes to keep the attachment system organized when confronted with distressing thoughts and feelings (Bowlby, 1980; George and West, 2012). As a result, they might become alert to these traumatic feelings and thoughts that cannot be successfully excluded from the consciousness and thus lead to a dysregulation during the attachment interview.

CV arousal also differed by attachment representation. The rise of SBP was by far highest in hypertensive patients with a secure attachment representation, even when controlled for potential confounders. This is in contrast to the results of Gallo and Matthews (2006) who showed a positive correlation between BP responses and insecure anxious and avoidant attachment (Feeney and Kirkpatrick, 1996; Gallo and Matthews, 2006). Importantly, they investigated normotensive individuals, while our study population suffered from severe hypertension treated with multiple anti-hypertensive agents. It is known that in the time course of clinical manifestation of hypertension, hemodynamic states change from an initially sympathetic over-activation with increased cardiac output and HR to normal cardiac output but high peripheral resistance in the later disease stages (Amerena and Julius, 1995; Schlaich et al., 2004). Healthy insecure anxious and avoidant attached individuals may respond initially by chronic sympathetic over-activation with increased BP responses (borderline hypertension). At later disease stages of hypertension, peripheral resistance increases due to microvascular remodeling, while physiological BP response to stressors, e.g., attachment related stress, is diminished. In populations with known coronary artery disease or at high risk for coronary artery disease due to hypertension and other risk factors, impaired increases in SBP are associated with poor CV outcome, while a higher SBP reactivity is associated with lower mortality (Naughton et al., 2000). Our sample represents a high-risk population, as all patients have diagnosed hypertension, and approximately half of them have a history of myocardial infarction. Therefore, the result that secure patients have a higher rise of SBP in our context of a high-risk population pinpoints toward a better prognosis of this subgroup. However, as RPP is a better index of myocardial oxygen consumption than SBP alone (Gobel et al., 1977), and as we found no significant differences in RPP between attachment groups nor in DBP, questions of CV reactivity and prognosis between attachment groups cannot be answered finally by our study. Further studies are needed to elucidate the ways in which attachment might contribute to the maintenance of hypertension.

Furthermore, we found differences in baseline HR among the different attachment groups with elevated HR in dismissing and unresolved hypertensive participants. HR is an independent risk factor for mortality (Hjalmarson, 2007). Though others found no baseline differences between attachment classifications (Stanley, 2006; Ablow et al., 2013), our results are in line with attachment theory. Dismissing individuals are those with highest repression of emotions (Dozier and Kobak, 1992), and individuals with unresolved trauma are unable to manage their emotions (Kobak and Madsen, 2008). This corroborates the hypothesis that in situations in which their defense mechanisms are overextended, dismissing individuals cannot longer regulate sufficiently their physiological arousal (Beijersbergen et al., 2008).

Next to the hypothesis that insecure attachment might prone the CV system toward hypertension via an altered stress response, a behavioral factor might contribute to our pronounced overrepresentation of patients with preoccupied attachment. A positive correlation between the number of visits at the general practitioner and preoccupied attachment, but not with secure or dismissing attachment has been shown in a study using AAI as attachment measure (Waller et al., 2004). Similar results were obtained using self-report measures (Ciechanowski, 2002; Meng et al., 2015). In most of the cases, hypertension is asymptomatic and is an incidental finding while consulting the physician for other reasons. Thus, the probability to be diagnosed with hypertension rises as health care utilization increases. This might contribute to the high proportion of preoccupied attachment in our sample of patients with diagnosed hypertension.

### Strengths and limitations

Our study is the first to investigate attachment representation and CV responses during the AAP in a hypertensive population. Our most important strength is our hypertensive population since it has been criticized that previous research on the influence of psychosocial factors on hypertension included exclusively samples with borderline or white-coat hypertension (Mann, 2012). In contrast to them, our study population consists of patients in a wide age range with clinically relevant hypertension, treated with multiple drugs, partly already affected by comorbidities developed in the consequence of their increased CV risk profile. Further strengths of our study are the accuracy of BP measurement, as we measured brachial BP which is recommended for clinical use with two sequential measurements for resting BP and measurements at both arms (Mancia et al., 2013). We also collected and reported important medical covariates, for example comorbidities and anti-hypertensive medication.

Another important strength is the use of the AAP interview to assess attachment representation instead of a self-report instrument. As this is an instrument which has proven to stimulate attachment (Buchheim et al., 2006, 2012), we were able to measure specifically attachment-related stress. It could be argued that the Thematic Apperception Test (TAT, Murray, 1943) would have been an interesting alternative since it is also a projective measure. The TAT is a widely used projective measure of personality and was assessed in several clinical samples to analyze the underlying dynamics of personality features from a psychodynamic perspective (e.g., Lysaker et al., 2007; Conway et al., 2013; Haggerty et al., 2015). In psychosomatic research, the TAT was e.g., used for a better understanding of the psychodynamics in patients with the chronic idiopathic pain syndrome (Sivik and Hösterey, 1992). A recent study used the TAT in combination with other projective measures to offer a comprehensive approach to personality assessment in a case of chronic depression (Husain, 2015). Although the TAT stories are likely interesting with respect to attachment, the TAT scenes were not generated by attachment theory, but from a clinical perspective to explore the underlying dynamics of personality in individuals. Many of the TAT drawings consist of sets of themes such as: success and failure, competition, and jealousy, feeling about relationships, aggression, and sexuality. In our study, we intended to activate the individual's attachment system and to assess the internal working models of attachment and therefore decided to use the AAP (George and West, 2012). As mentioned in the Materials and Methods Section, the pictures of the AAP are designed to elicit attachment distress depicting the most prominent attachment activators in the Bowlby–Ainsworth approach, such as separation, solitude, fear, and death. Moreover, the pictures capture variations in the perceived accessibility of attachment figures. Attachment figure availability, combined with responsive and effective care, is central to internal working models of attachment security. Our results indicate that this measure was appropriate to use by taking into account the specific goals of our study.

The results of this study need to be interpreted in the light of the following limitations. Firstly, our sample is not representative, as we recruited the participants solely in a cardiologic outpatient clinic of a University hospital. Moreover, our sample size is very small, especially the subgroup of female patients. However, smaller samples are common in narrative-based research, especially in clinical groups (Ravitz et al., 2010), as the interview procedure is time-consuming in administration and coding. Nevertheless, our results should therefore be treated with caution and need further investigation using larger sample sizes. In addition, we also excluded patients with trauma-related symptoms. Due to the exclusion of patients with a possible PTSD, the prevalence rate of unresolved trauma that we found in our sample might be underestimated. Secondly, we did not assess depression and other mental illnesses in our sample using a standardized clinical interview. Lastly, interpretation of CV responses to the AAP is limited by the fact that we had no control session with emotion- and attachment-neutral content, which we plan to include in future studies.

## Conclusion

In conclusion, our results demonstrate that insecure attachment is highly prevalent in patients with primary hypertension and almost twice as frequent as in non-clinical individuals (Bakermans-Kranenburg and Van IJzendoorn, 2009). We showed a physiological arousal during attachment activating stimuli in these patients. Our results strengthen the potential association between insecure attachment and hypertension, but also show that pathophysiological mechanisms need to be focused in more detail. For clinicians, it is important to notice the high amount of insecure attachment in hypertensive patients as this might be an important factor influencing compliance (Ciechanowski et al., 2001; Aarts et al., 2015).

## Author contributions

EB designed the study, supervised data acquisition and conduction of the experiments, performed the statistical analyses, interpreted the data and drafted the manuscript. MG analyzed part of the attachment interviews, interpreted the data and drafted the manuscript. DP performed the statistical analyses, interpreted the data and critically revised the manuscript for important intellectual content. AF was in charge of data acquisition and conduction of the experiments and critically revised the manuscript for important intellectual content. CW designed the study, contributed to the conception of the experimental setting, supervised the study, interpreted the data and critically revised the manuscript for important intellectual content. AB contributed to the conception of the experimental setting, supervised the study, analyzed part of the attachment interviews, interpreted the data and critically revised the manuscript for important intellectual content. All authors approved the final version of the manuscript to be published and agree to be accountable for all aspects of the work in ensuring that questions related to the accuracy or integrity of any part of the work are appropriately investigated and resolved.

### Conflict of interest statement

The authors declare that the research was conducted in the absence of any commercial or financial relationships that could be construed as a potential conflict of interest.

## Abbreviations

AAP: Adult Attachment Projective picture system
AAI: Adult Attachment Interview
BP: Blood Pressure
CI: Confidence Interval
CV: CardioVascular
DBP: Diastolic Blood Pressure
HR: Heart Rate
MDBF: Multidimensional Mood Questionnaire (MehrDimensionaler Befindlichkeits-Fragebogen)
PTSD: Post-Traumatic Stress Disease
RPP: Rate-Pressure Product
SBP: Systolic Blood Pressure
SD: Standard Deviation
STAI-S: State Trait Anxiety Inventory-State
TAT: Thematic Apperception Test.
